# Cytosolic NUAK1 Enhances ATP Production by Maintaining Proper Glycolysis and Mitochondrial Function in Cancer Cells

**DOI:** 10.3389/fonc.2020.01123

**Published:** 2020-07-10

**Authors:** Emilia Escalona, Marcelo Muñoz, Roxana Pincheira, Álvaro A. Elorza, Ariel F. Castro

**Affiliations:** ^1^Signal Transduction and Cancer Laboratory, Biochemistry and Molecular Biology Department, Faculty of Biological Sciences, Universidad de Concepción, Concepción, Chile; ^2^Mitochondrial Medicine Laboratory, Institute of Biomedical Sciences, Faculty of Medicine and Faculty of Life Sciences, Universidad Andres Bello, Santiago, Chile

**Keywords:** NUAK1, cancer metabolism, cell bioenergetic, oxidative cells, glycolytic switch, seahorse assay, mitochondrial donut

## Abstract

NUAK1 is an AMPK-related kinase located in the cytosol and the nucleus, whose expression associates with tumor malignancy and poor patient prognosis in several cancers. Accordingly, NUAK1 was associated with metastasis because it promotes cell migration and invasion in different cancer cells. Besides, NUAK1 supports cancer cell survival under metabolic stress and maintains ATP levels in hepatocarcinoma cells, suggesting a role in energy metabolism in cancer. However, the underlying mechanism for this metabolic function, as well as its link to NUAK1 subcellular localization, is unclear. We demonstrated that cytosolic NUAK1 increases ATP levels, which associates with increased mitochondrial respiration, supporting that cytosolic NUAK1 is involved in mitochondrial function regulation in cancer cells. NUAK1 inhibition led to the formation of “donut-like” structures, providing evidence of NUAK1-dependent mitochondrial morphology regulation. Additionally, our results indicated that cytosolic NUAK1 increases the glycolytic capacity of cancer cells under mitochondrial inhibition. Nuclear NUAK1 seems to be involved in the metabolic switch to glycolysis. Altogether, our results suggest that cytosolic NUAK1 participates in mitochondrial ATP production and the maintenance of proper glycolysis in cancer cells. Our current studies support the role of NUAK1 in bioenergetics, mitochondrial homeostasis, glycolysis and metabolic capacities. They suggest different metabolic outcomes depending on its subcellular localization. The identified roles of NUAK1 in cancer metabolism provide a potential mechanism relevant for tumor progression and its association with poor patient prognosis in several cancers. Further studies could shed light on the molecular mechanisms involved in the identified metabolic NUAK1 functions.

## Introduction

Cancer metabolism has become a trending topic in cancer research because it participates in tumorigenesis and cancer progression. Tumor cells are continuously exposed to wide metabolic changes, such as nutrient starvation, hypoxia, and microenvironment acidification (1, 2). Thus, tumor progression success depends on the capacity of cancer cells to adapt and surpass this metabolic challenge (2). Understanding the mechanisms and proteins involved in cancer cell's metabolic changes is critical for the development of new therapies.

NUAK1 is a serine/threonine kinase related by sequence homology to the catalytic α-subunits of the metabolic regulator AMPK (3). Multiple cancers overexpress NUAK1, such as hepatocarcinoma (4), colon cancer (5), glioma (6), and breast cancer within others (7, 8). NUAK1 shows stage-dependent expression in cancer tissues and associates with tumor malignancy and poor patient prognosis (6, 9–11). According to its association with cancer, NUAK1 plays a role in several processes related to tumor progression, including cell migration (12), invasion, and metastasis (13).

Additionally, NUAK1 plays a role in the survival of cancer cells (14, 15), protecting them from cell death induced by oxidative or metabolic stress. NUAK1 protected from metabolic stress through maintaining energy balance in MYC-driven cancer cells, which were unable to balance ATP levels, and mitochondrial function in the absence of NUAK1 (16). Also downstream of MYC, Calcium/PKCα-dependent activation of NUAK1 supported cell survival by engaging the AMPK-mTORC1 metabolic checkpoint (17). NUAK1 association with metabolism and survival seems to be independent of p53, the most frequently mutated and inactivated gene in cancer (18). Although there is a report suggesting that NUAK1 can regulate the p53 transcription factor, knock-down of NUAK1 provoked loss of ATP and cell death in p53-null hepatoma cells (16). Thus, NUAK1 might also be relevant in metabolism and tumor progression in a p53-independent context.

We recently showed that NUAK1 has nuclear and cytosolic subcellular locations regulated by active nuclear transport (19). Others and our studies indicate that NUAK1 distribution is cell- and context-specific, and might be associated with the clinical stage of cancer, displaying a cytosolic accumulation in late-stages histopathological samples (6, 10). Thus, NUAK1 may have specific functions according its subcellular localization. Consistent with a nuclear-associated function, NUAK1 was recently involved in promoting spliceosome activity (20). In association with its effect on cell migration, the cytosolic NUAK1 phosphorylates the myosin phosphatase targeting-1 (MYPT1), promoting cell detachment (12). However, it is unknown how NUAK1's effect on metabolism associates with its subcellular localization.

Here, we show that cytosolic NUAK1 increases the cellular bioenergetic state, mainly associated with mitochondrial respiration maintenance. Additionally, perturbations on NUAK1 function affect mitochondrial morphology. NUAK1 also shows a role in glycolysis, particularly in its nuclear localization. Our work suggests that the subcellular localization of NUAK1 is relevant to its specific metabolic effects.

## Materials and Methods

### Cell Culture

Cancer cells lines HCT116 p53 null, kindly provided by Dr. B. Vogelstein (Johns Hopkins Medicine, USA) (21), and HeLa (ATCC® CCL-2™, Manassas, VA) were cultured in Dulbecco's modified Eagles's medium (DMEM) containing 4.5 g/l glucose, 2 mM L-glutamine, and 1 mM sodium pyruvate (Corning, New York, USA). MDA-MB-231 cells (ATCC® HTB-26) were cultured in DMEM containing 1 g/l glucose, 2 mM L-glutamine and 1 mM sodium pyruvate (HyClone, Logan, UT, USA) and MCF-7 cells (ATCC® HTB-22) were maintained in Minimal Essential Medium (MEM) with Earle's Balanced Salt Solution (EBSS) containing 1 g/l glucose, 2 mM L-glutamine and 1 mM sodium pyruvate (HyClone). All culture mediums were supplemented with 100 ug/ml streptomycin (HyClone), 100 U/ml penicillin (HyClone), 2.5 ug/ml Plasmocin (InvivoGen, San Diego, CA, USA), 10% fetal bovine serum (Biological Industries) and incubated at 37 °C in 5% CO_2_. *Mycoplasma-*free cultures were frequently tested with EZ-PCR Mycoplasma Kit (Biological Industries, CT, USA). The hypoxic environment (1% O_2_) was generated in a hypoxia chamber (STEMCELL Technologies, Vancouver, Canada).

### Cell Transfection

Cells were transfected using Lipofectamine 3000 (ThermoFisher, Waltham, MA, USA) or Lipofectamine 2000 (for HeLa cells). For the overexpression of wild type NUAK1 and a nuclear-deficient NUAK1 mutant (19), we used pCMV-FLAG-hNUAK1 and pCMV-FLAG-hNUAK1-KR43/70AA (NUAK1cyt) plasmids, respectively, and the pCMV-2-FLAG plasmid as control. The pLKO system was used to silence NUAK1 expression (22). The shRNA for NUAK1: 5′- TGGCCGAGTGGTTGCTATAAA-3′ was purchased from Sigma-Aldrich (St. Louis, MO, USA).

### Chemicals and Antibodies

Protease inhibitor and Phosphate inhibitor cocktails, 2-Deoxy-D-glucose, Oligomycin A, Carbonyl cyanide 4-(trifluoromethoxy)phenylhydrazone, Antimycin A and Rotenone were purchased from Sigma-Aldrich. HTH-01-015, a potent and selective NUAK1 inhibitor (23) was from Tocris (Bristol, UK). Fluorophores Tetramethylrhodamine Ethyl Ester, Perchlorate (TMRE), MitoTracker™ Green FM, and Hoechst 33,342 were from ThermoFisher. AccuRuler RGB plus protein ladder was purchased from MaestroGen Inc. (Hsinchu City, Taiwan). Anti-NUAK1 antibody (#4458) was from Cell Signaling (Danvers, MA, USA), and the anti-FLAG (M2) was from Sigma-Aldrich. Antibodies against β-Actin (AC-15), ATP5B (E-1), and TOM20 (F-10) were purchased from Santa Cruz Biotechnology (Dallas, TX, USA). Total OXPHOS Rodent WB Antibody Cocktail (ab110413) was from Abcam (Cambridge, United Kingdom). Goat Secondary antibodies anti-mouse IgG-HRP and anti-rabbit IgG-HRP conjugates were purchased from Bio-Rad (Hercules, CA, USA). The anti-mouse Alexa-488 antibody (A11001) was from ThermoFisher.

### Immunoblotting

Cell lines were lysed with modified NP-40 buffer (1%, NP-40, 25 mM Tris/HCl pH 7.4, 2.2 mM MgCl_2_, 1 mM EDTA, NaCl 150 mM, 5% Glycerol). Total proteins from lysates were fractionated by SDS–polyacrylamide gel electrophoresis and transferred to PDVF membranes. Finally, membranes were incubated 3 min with ECL Western Blotting Detection Reagent (GE Healthcare, Amersham, UK). Immunolabeled proteins were visualized in Syngene PXi6 Documentation System (Frederick, MD, USA).

### Immunofluorescence Microscopy

Cells grown on coverslips were prepared as previously described (19). After incubation with the FLAG and the secondary anti-mouse Alexa-488 antibodies, images were obtained with an LMS 780 spectral confocal system (Zeiss, Jena, Germany).

### ATP Measurement

An equal number of cells were seeded in 96-well plates and 16 h later transfected. Twenty-four hours post-transfection of cells, ATP was measured by using the ATP Determination Kit (Invitrogen) according to the manufacturer's protocol. ATP levels were expressed as the percentage of their control group (arbitrary set to 100%) and normalized to the corresponding protein concentration.

### Oxygen Consumption Rate (OCR) and Extracellular Acidification Rate (ECAR)

The mitochondrial respiratory activity and glycolysis status of live cells were measured by detection of cellular oxygen consumption rate (OCR) and extracellular acidification rate (ECAR), using a Seahorse XF24 (Agilent, Santa Clara, CA, USA). Briefly, 3 x 10^4^ MCF-7 or MDA-MB-231 cells per well were plated on the XF24 culture plate and incubated at 37°C in 5% CO_2_. The following day, the cells were incubated 1 h at 37 °C without CO_2_ and washed 3 times with seahorse medium containing phenol red-free DMEM base (D5030, Sigma-Aldrich), 2 mM L-glutamine, and 1 mM pyruvate. The XF24 culture plate plus the cartridge pre-incubated with Seahorse XF Calibrant Solution (103059-000, Agilent) were mounted in the analyzer. OCR was recorded as pmolO_2_/min, and ECAR was recorded as mpH/min. On the course of the assay, four sequential injections were performed after three readings in order to analyze OXPHOS and glycolytic parameters. For MCF-7 cells, it was sequentially injected to final concentration 5.5 mM glucose, 1.2 μM Oligomycin A, 0.5 μM FCCP, and finally 2 μM Rotenone with 2 μM Antimycin A. For MDA-MB-231 cells, it was used 5.5 mM glucose, 1 μM Oligomycin A, 1 μM FCCP, 1 μM Rotenone, and 1 μM Antimycin A. Right after the assay is ended, OCR and ECAR from each sample were normalized to the corresponding total protein concentration before calculation of metabolic parameters. Briefly, cells were lysed with SDS Lysis Buffer containing 20 mM HEPES, 2 mM EDTA, 0.5% Triton X-100, 0.1% SDS and 1 mM PMSF at 4°C. Proteins concentration was quantified by Bradford method. For OXPHOS parameters, we used normalized OCR and set the Non-Mitochondrial Oxygen Consumption (Non-MOC) as the minimum rate after Rotenone/antimycin A injection. Basal respiration was the last rate after glucose injection minus the Non-MOC; Maximal respiration was the maximum rate after FCCP injection minus the non-MOC; Proton Leak was the minimum rate after oligomycin injection minus the Non-MOC. ATP Production Coupled Respiration was the last rate before oligomycin injection minus the minimum rate after oligomycin injection, and Spare Respiratory Capacity was the maximal respiration minus the basal respiration. To analyze glycolysis, we used normalized ECAR. Glycolysis parameter was the maximum rate before oligomycin injection minus the last rate before glucose injection. Glycolytic capacity was the maximum rate after oligomycin injection minus the last rate before glucose injection, and glycolytic reserve was the glycolytic capacity minus the glycolysis parameter. All assays were done in triplicate and repeated three times.

### Lactate Measurement

HCT116 p53-null cells (3.5 x 10^5^) were plated in a 24-well plate and incubated overnight at 37 °C in 5% CO_2_. The medium was replaced for phenol red-free medium, and cells were treated for 24 h. For recovering extracellular lactate, the supernatant was mixed with trichloroacetic acid 0.6N in 1:2 proportion in ice, mixed for 30 s, incubated at 4°C for 5 min and interfering proteins were precipitated and removed by centrifugation at 1500xG. Extracellular lactate concentration was calculated by measurement of NADH product obtained by a coupled-enzymatic method using L-lactate dehydrogenase (Sigma). NADH was detected by absorbance at 340 nm. Simultaneously, the cells were lysed with SDS Lysis Buffer, and protein concentration was measured by Bradford method. Results were expressed as μM lactate/μg protein. All assays were done in triplicate.

### *In vivo* Microscopy

Cells were plated in a 35 mm imaging dish and incubated overnight at 37 °C in 5% CO_2_. For the nuclear and mitochondrial staining, the cells were incubated with 200 nM Mitotracker Green and 5 μg/ml Hoechst 33,342 for 30 min in PBS at 37 °C in 5% CO_2_. After washed the cells twice with PBS, 10 mM TMRE was added in a fresh culture medium. For the *in vivo* microscopy, the cells were maintained in the Chamlide chamber (Lice cell instrument, Seoul, Korea) and images were capture using Olympus FV1000 microscopy (Center Valley, PA, USA). The Z-stack was transformed into maximal intensity projection. The image analysis was performed using ImageJ software (24).

### Statistical Analysis

Statistical analysis and graphics were performed with GraphPad Prism 6. Statistical significance was determined by unpaired Student's *t*-test or two-way ANOVA with Holm-Sidak correction. Differences were considered statistically significant if *p* < 0.05.

## Results

### Cytosolic NUAK1 Enhances Cellular ATP in Cancer Cells

**Figure 1:**
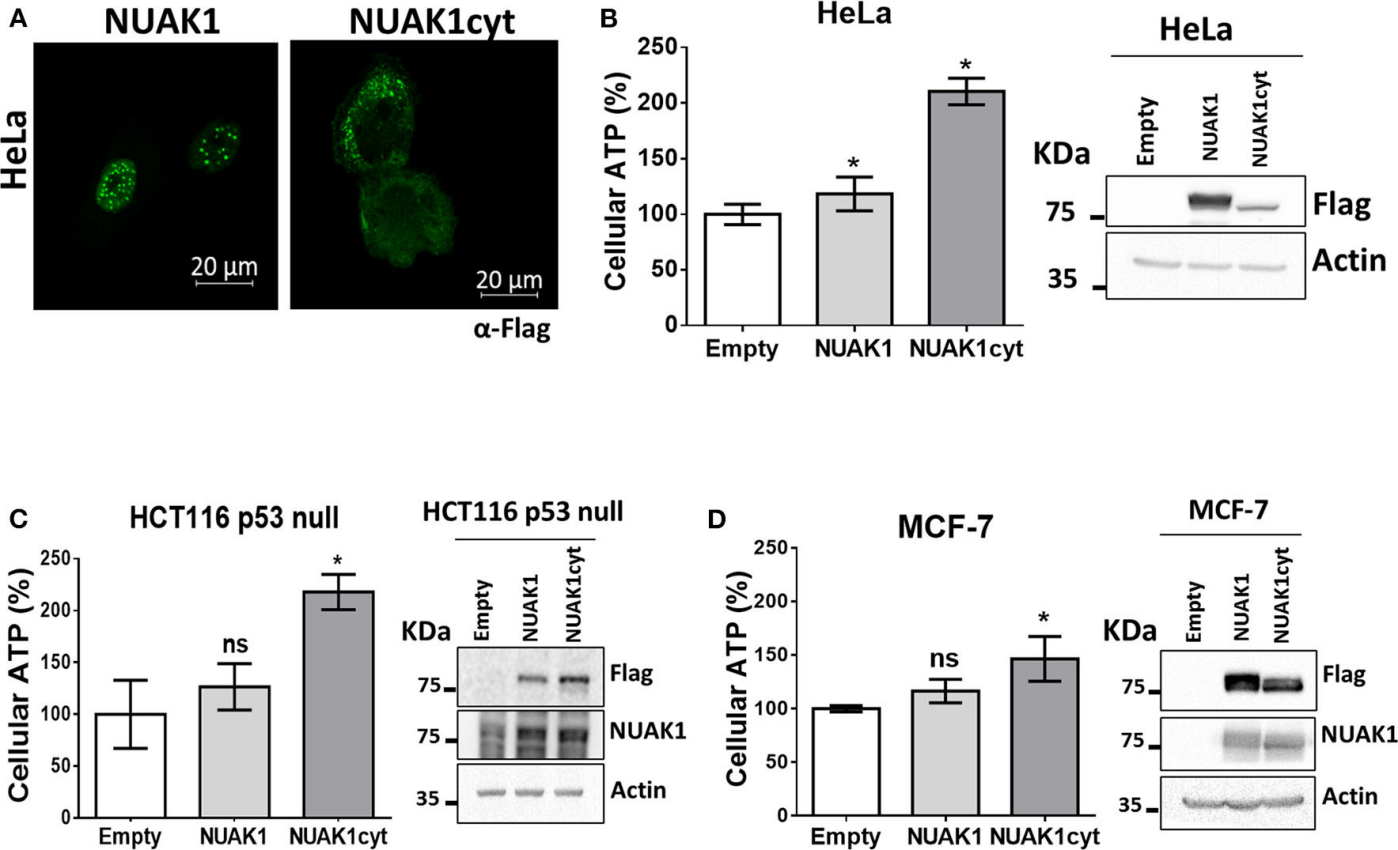
Cytosolic NUAK1 increases cellular ATP in cancer cells. **(A)** Immunocytochemistry images of NUAK1 location in HeLa cells expressing FLAG-NUAK1 WT or FLAG-hNUAK1-KR43/70AA (NUAK1cyt) mutant. Cells were stained with FLAG-antibody, 630X zoom. ATP levels in **(B)** HeLa, **(C)** HCT116 p53-null and **(D)** MCF-7 cells 24 h post-transfection with FLAG-NUAK1 WT (gray bar) or FLAG-NUAK1cyt mutant vector (dark gray bar). Empty vector was used as control (white bar) and results were expressed as a percentage relative to the control group. The results are representative of three independent experiments (*n* = 3). Each bar represents the mean ± S.D, *p < 0.05. On the right, immunoblots showing NUAK1 expression. NUAK1 was detected with FLAG antibody or a specific antibody against NUAK1. Actin was used as the loading control.

We have previously found that endogenous NUAK1 has a diverse subcellular localization depending on the cancer cell line, mostly located in the nucleus or the cytoplasm, or with an equilibrated distribution (19). From these previous studies, we choose cancer cells with high nuclear NUAK1 expression (HeLa and HCT116 p53-null cells) or with high cytosolic expression (MCF-7 cells). To initially investigate whether the metabolic function of NUAK1 associates with a specific subcellular location, we used a previously characterized nuclear-deficient NUAK1 mutant, from now on cytosolic NUAK1. Like the endogenous NUAK1 (19), immunocytochemistry assay showed that overexpressed wild type NUAK1 was mainly in the nucleus of HeLa cells, while the cytosolic NUAK1 showed the expected location (Figure 1A). Both wild type and cytosolic NUAK1 significantly increased ATP levels in HeLa cells; however, the cytosolic NUAK1 induced a higher ATP increment (Figure 1B). We found that only the cytosolic NUAK1 increases ATP levels in the colon HCT 116 p53-null cancer cells (Figure 1C), where endogenous NUAK1 is not detected in the cytoplasm (19). Although MFC-7 cells have high endogenous cytosolic NUAK1 expression (19), the expression of the cytosolic NUAK1 mutant could further increase the ATP levels, although to a lesser extent (Figure 1D). Altogether, our results suggest that the cytosolic NUAK1 associated with cancer cell bioenergetics.

### NUAK1 Affects Mitochondrial Respiration Parameters and Mitochondrial Membrane Potential

**Figure 2:**
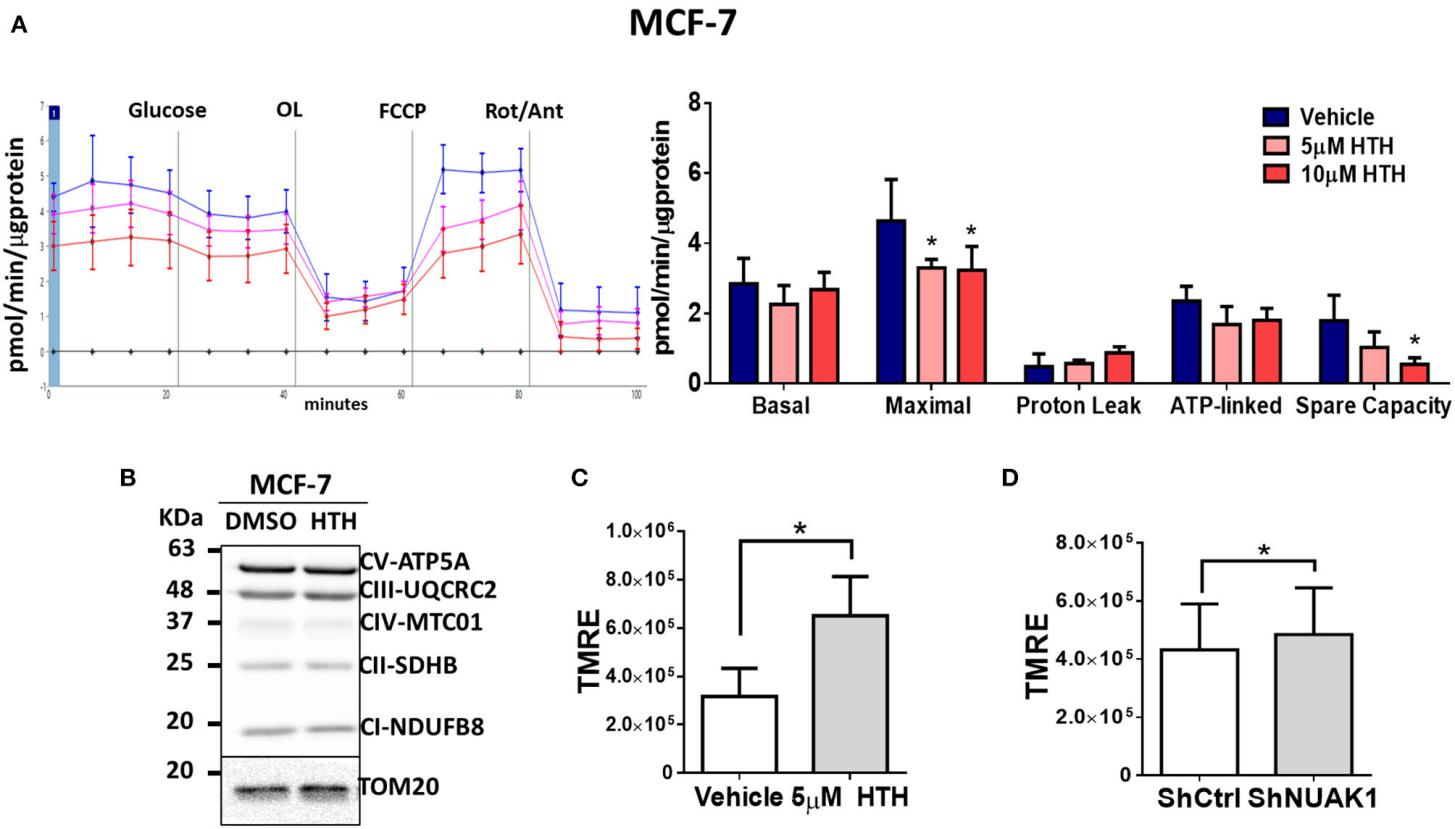
NUAK1 allows proper mitochondrial function in breast cancer cells. **(A**,Left**)** Oxygen consumption rates of MCF-7 cells after 4 h incubation with 5 μM (pink curve) or 10 μM (red curve) HTH-01-015, or DMSO used as vehicle (blue curve), measured using the Seahorse XF24. At the times indicated, glucose, oligomycin (OL), FCCP, and Rotenone (Rot) with Antimycin A (Ant) were injected as described in the Methods section. (Right) Respiration parameters from the experiments on the *left*. Graph shows respiration parameters from MCF-7 cells with 5 μM (pink bar) or 10 μM (red bar) HTH-01-015, or DMSO (blue bar). All values were normalized to the corresponding protein concentration. OCR average ± SD from three independent experiments, *P < 0.05. **(B)** Western blot showing OXPHOS complex proteins from MCF-7 cells after 4 h incubation with 5 μM HTH-01-015 or DMSO. TOM20 was used as the loading control. **(C)** Quantification of TMRE mean intensity from *in vivo* microscopy of MCF-7 cells treated for 4 h with 5 μM HTH-01-015 or vehicle (*n* = 80), *P < 0.05. **(D)** Quantification of TMRE mean intensity from *in vivo* microscopy of NUAK1-silenced MCF-7 cells and control group (*n* = 80), *p < 0.05.

The increase in cellular ATP could be due to alterations on either ATP consumption or ATP production. To discern between these two processes, we examined the mitochondrial responses by measuring the OCR under normal conditions or stimulation with pharmacological mitochondrial modulators. According to the above results, for these assays, we used MCF-7 cancer cells because they depend more on mitochondrial function for their bioenergetics demands (25). In addition, MCF-7 cells have high cytosolic NUAK1 expression, which is suitable to infer the role of the endogenous NUAK1. We used 10 μM HTH-01-015, a selective NUAK1 kinase inhibitor (23, 26). We found that NUAK1 inhibition significantly decreased maximal respiration (FCCP-stimulated) in MCF-7 cells; still, HTH-01-015 affected mitochondrial spare respiratory capacity, but no other mitochondrial respiration parameters (Figure 2A). The decrease in maximal respiration by NUAK1 inhibition was not accompanied by changes in mitochondrial protein expression (Figure 2B). Supporting that NUAK1 activity affects mitochondrial function, HTH-01-015 treatment (Figure 2C) and shRNA-mediated knock-down of NUAK1 expression (Figure 2D) significantly increased the mitochondrial membrane potential (mtΔΨ).

**Figure 3:**
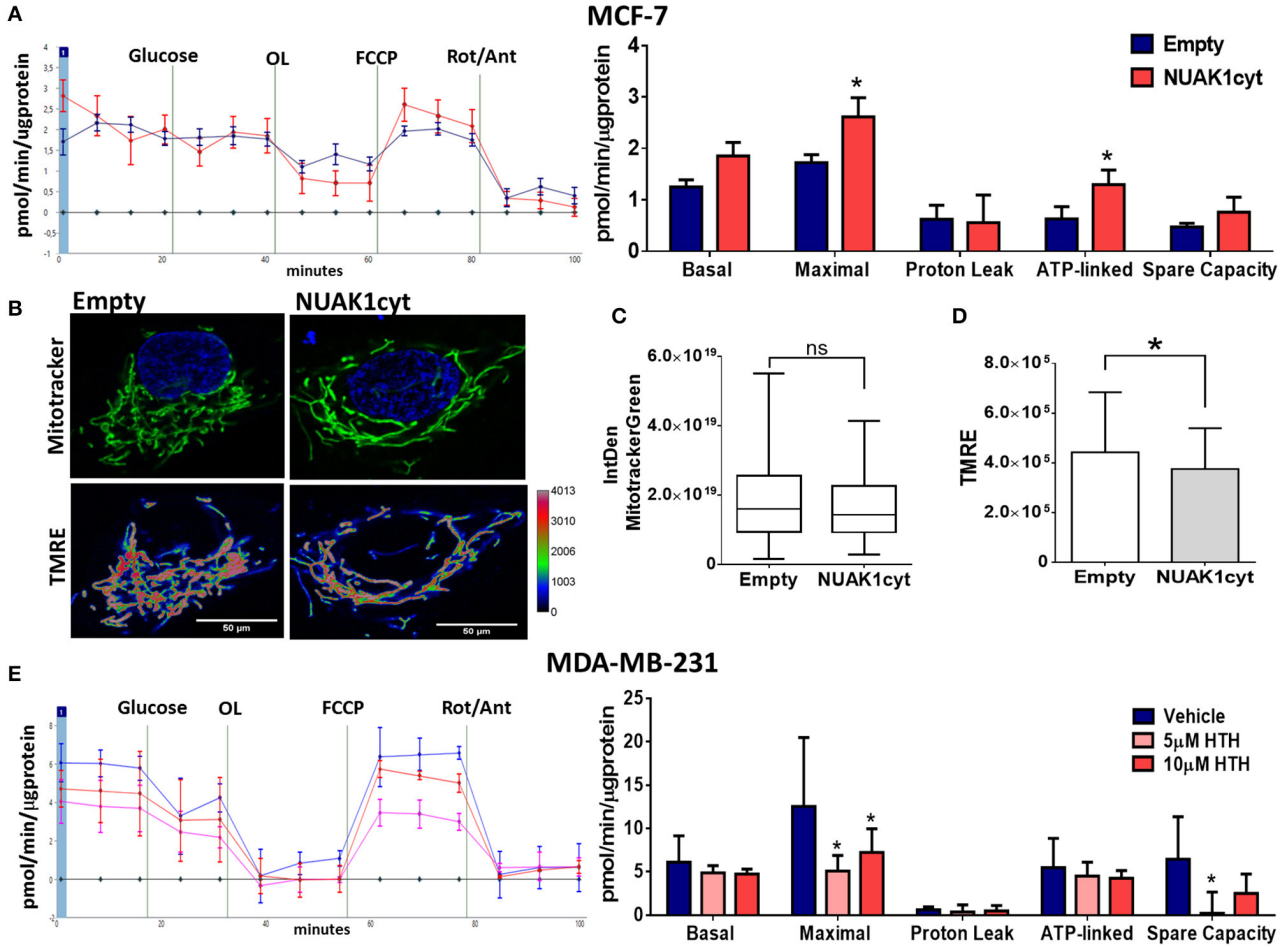
Cytosolic NUAK1 expression is involved in mitochondrial function regulation in breast cancer cells. **(A**, Left**)** Oxygen consumption rates of MCF-7 cells 24 h post-transfection with FLAG-NUAK1cyt mutant (red curve) or empty vector (blue curve), measured using the Seahorse XF24. At the times indicated, glucose, oligomycin (OL), FCCP, and Rotenone (Rot) with Antimycin A (Ant) were injected as described in the Methods section. (Right) Respiration parameters from the experiments on the *left*. Graph shows respiration parameters from MCF-7 cells transfected with FLAG-NUAK1cyt mutant (red bar) or empty vector (blue bar). All values were normalized to the corresponding protein concentration. OCR average ± SD from 3 independent experiments, *p < 0.05. **(B)** *in vivo* microscopy images of MCF-7 cells expressing FLAG-NUAK1cyt mutant or empty vector stained with mitotracker green (green), TMRE and Hoechst for nuclei (blue). Fluorescence intensity of TMRE is represented in pseudo color scale (“Rainbow RGB” in ImageJ software). 600X optical zoom plus 3X digital zoom. **(C)** Quantification of mitotracker green integrated density (*n* = 90). *p < 0.05. **(D)** Plots of TMRE mean intensity quantification (*n* = 90). *p < 0.05. **(E)** Same as in **(A)** for MDA-MB-231 cells. (Left) Oxygen consumption rates of MDA-MB-231 cells with 5 μM (pink curve) or 10 μM (red curve) HTH-01-015, or DMSO (blue curve). (Right) Respiration parameters from the experiments on the left. Graph shows respiration parameters from MDA-MB-231 cells with 5 μM (pink bar) or 10 μM (red bar) HTH-01-015, or DMSO (blue bar). All values were normalized to the corresponding protein concentration. OCR average ± SD from three independent experiments, *p < 0.05.

Then, we analyzed the association of the increase of cellular ATP by the cytosolic NUAK1 in MCF-7 cells with mitochondrial respiration. According to the above results, the cytosolic NUAK1 increased maximal respiration (Figure 3A) and significantly decreased the mtΔΨ (Figures 3B,C), indicating that cytosolic NUAK1 induces ATP synthase activity (oligomycin-insensitive respiration showed no NUAK1-induced leaking). Besides, no significant changes in mitochondrial volume were observed (Figure 3D). To further confirm the role of the cytosolic NUAK1 in breast cancer cells, we used MDA-MB-231 cells, where NUAK1 only detected in the cytosolic fraction (19). Accordingly, we also found that NUAK1 inhibition significantly decreases maximal mitochondrial respiration and spare mitochondrial capacities (Figure 3E). In agreement with an exclusive cytosolic location of NUAK1 in MDA-MB-231 cells, the maximal respiration parameter was much higher than in MCF-7 cells and was strongly affected by NUAK1 inhibition. Altogether, our data suggest that the cytosolic NUAK1 enhances breast cancer cell bioenergetics by increasing the mitochondrial respiratory capacity.

### The Downregulation of NUAK1 Induces Mitochondrial Morphology Alterations

**Figure 4:**
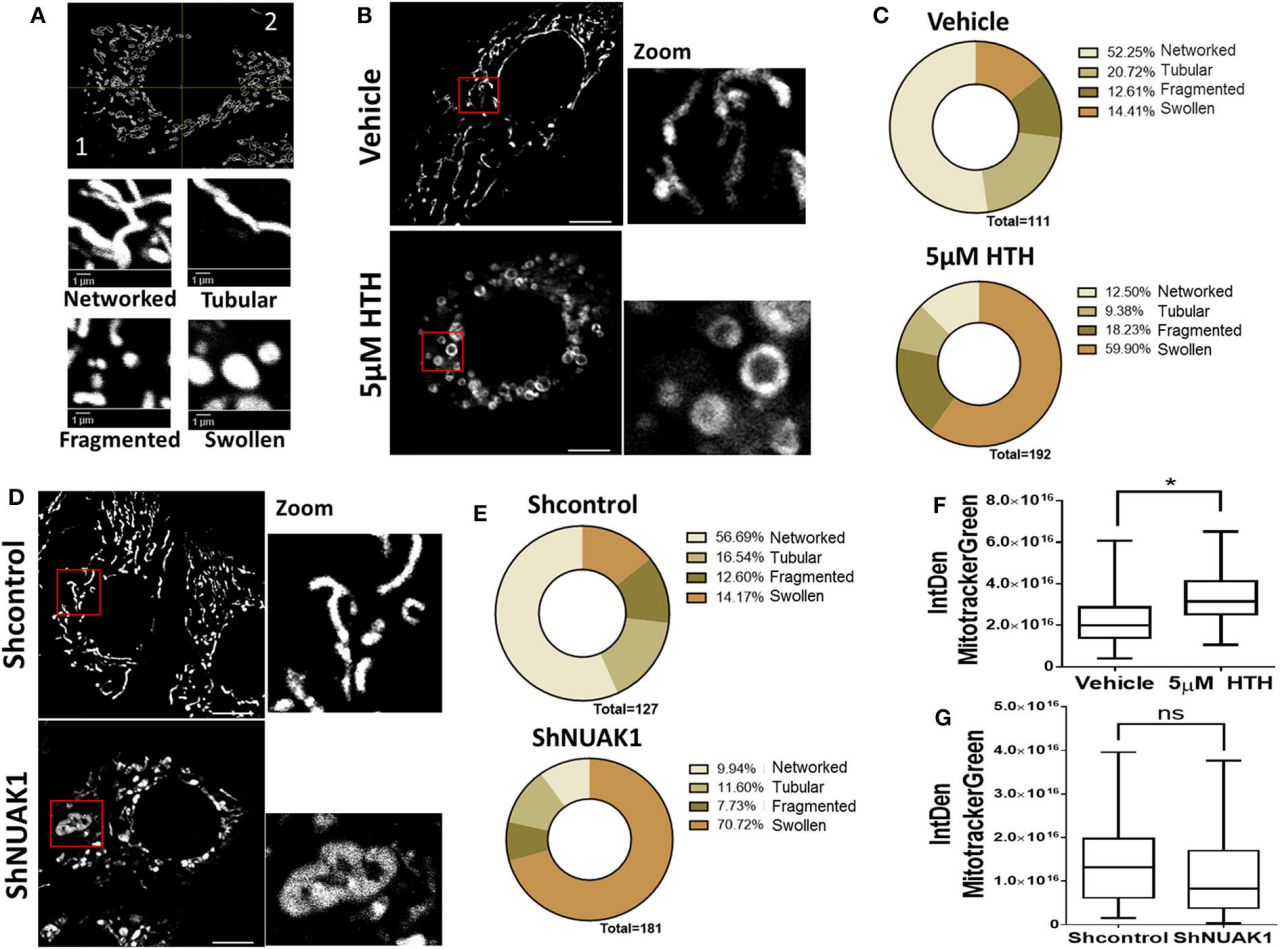
Downregulation of N UAK1 function induces mitochondrial morphology alterations in MCF-7 cells. **(A)** *in vivo* microscopy of MCF-7 cells treated for 4 h with 5 μM HTH-01-015 or vehicle, stained with TMRE (red) and mitotracker green (green). Scale bar equal to 10 μm. 600X optical zoom plus 5X digital zoom. **(B)** Quantification of mitotracker green integrated density of MCF-7 cells treated with 5 μM HTH-01-015 or vehicle (*n* = 80), *p < 0.05. **(C)** Representative image used for mitochondrial morphology quantification. Mitochondria were classified in networked, tubular, fragmented and swollen from two quadrants. **(D)** Mitochondrial morphology quantification of MCF-7 cells treated for 4 h with 5 μM HTH-01-015 and control cells. **(E)** *in vivo* microscopy of NUAK1 depleted MCF-7 cells and control group stained with TMRE (red) and mitotracker green (green). Scale bar equal to 10 μm. 600X optical zoom plus 5X digital zoom. **(F)** Quantification of mitotracker green integrated density of NUAK1 depleted MCF-7 cells and control cells (*n* = 80), *p < 0.05. **(G)** Mitochondrial morphology quantification of NUAK1 depleted MCF-7 cells and control cells (*n* = 80), *p < 0.05.

Mitochondria are dynamic organelles, and their structures frequently reflect bioenergetics state or dysfunctions. Thus, to understand the NUAK1 function on mitochondria, we additionally investigated whether it affects mitochondria morphology, identifying networked, tubular, fragmented, and large and round mitochondria (Figure 4A). We observed that HTH-01-015 treatment drastically changed the mitochondria morphology of MCF-7 cells, from mainly networked and tubular to large and round mitochondrial structure (Figures 4B,C). Interestingly, mitotracker green images showed a mitochondrial structure known as “donut” rather than the typical punctate mitochondrion (Figure 4B). To validate that our morphological observations were specifically associated with the inhibition of NUAK1, we knocked-down NUAK1 in MCF-7 cells and performed mitochondria morphology analysis. The knock-down of NUAK1 also changed the mitochondrial morphology from networked to large and round shape (Figures 4D,E), but we observed less “donut” structures than the treatment with the inhibitor (Figure 4D). On the other hand, the overexpression of the cytosolic NUAK1 showed no significant impact on mitochondrial morphology (Figure 2B). These data suggest that NUAK1 activity maintains a suitable mitochondrial morphology. Interestingly, NUAK1 inhibition by HTH-01-015 showed a significant increase in mitochondrial volume (Figure 4F), whereas the NUAK1 knock-down showed no significant differences between groups (Figure 4G). This apparent discrepancy may be related to differences in the time points for the volume evaluation, measured at 4 h after HTH-01-015 treatment or at 24 h in NUAK1 knock-down cells. Summarizing, our data showed that NUAK1 is necessary for maintaining proper mitochondrial morphology in MCF-7 cells.

### NUAK1 Is Involved in Glycolytic Capacity Regulation

**Figure 5:**
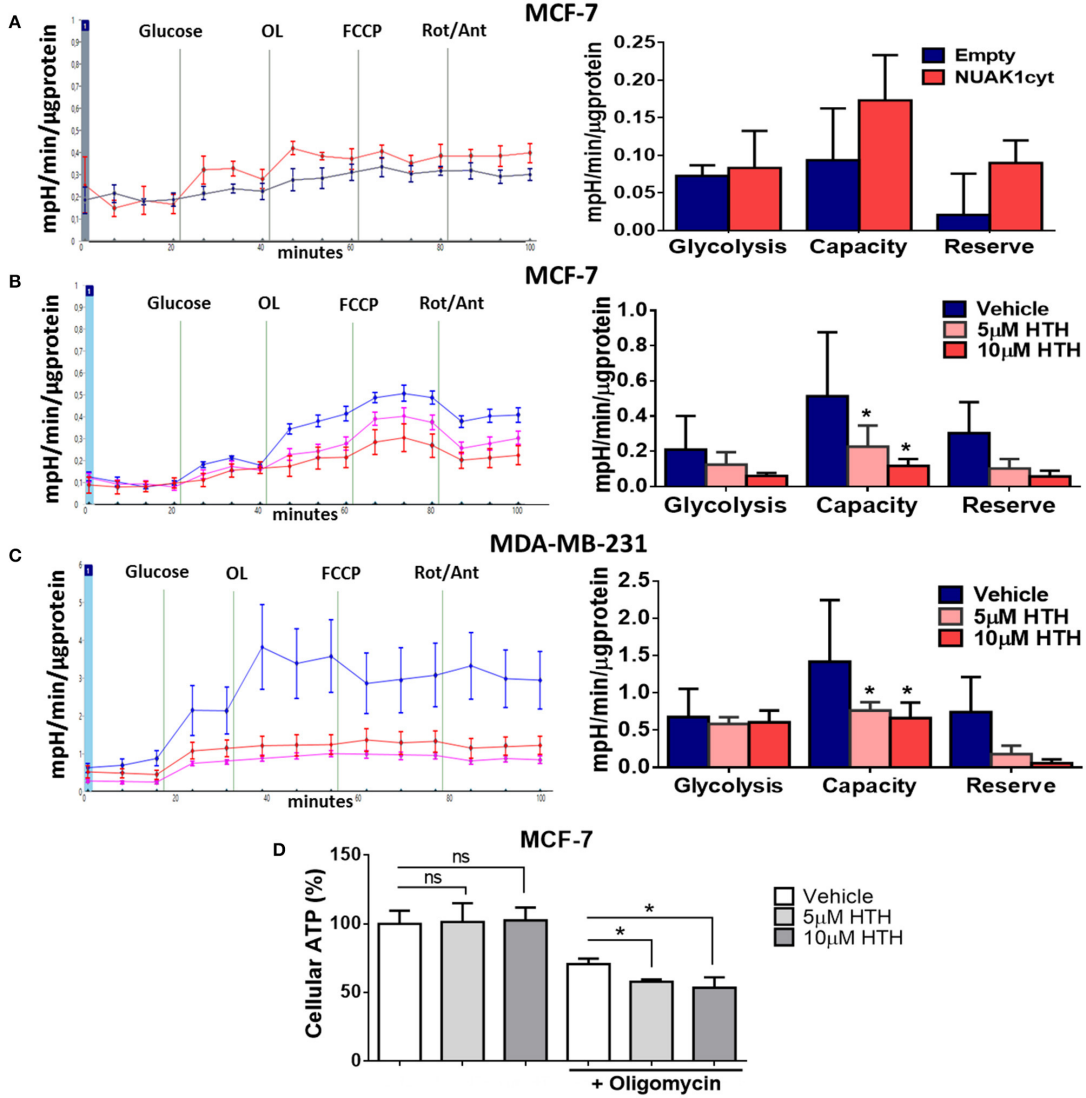
Cytosolic NUAK1 plays a role in the regulation of glycolysis in breast cancer cells. **(A**, Left**)** Kinetic of extracellular acidification from MCF-7 cells 24 h post-transfection with FLAG-NUAK1cyt mutant (red curve) or empty vector (blue curve), measured using the Seahorse XF24. At the times indicated, glucose, oligomycin (OL), FCCP, and Rotenone (Rot) with Antimycin A (Ant) were injected as described in the Methods section. (Right) Glycolytic parameters evaluation from the experiments on the *left*. Graph shows MCF-7 cells with FLAG-NUAK1cyt mutant (red bar) or empty vector (blue bar). **(B)** Same as in **(A**, Left**)**. Kinetic of extracellular acidification from MCF-7 cells after 4 h of treatment with 5 μM HTH-01-015 (pink curve), 10 μM HTH-01-015 (red curve) or vehicle (blue curve). Right. Glycolytic parameters evaluation from the experiments on the *left*. Graph shows cells with 5 μM HTH-01-015 (pink bar), 10 μM HTH-01-015 (red bar) or vehicle (blue bar). **(C)** Same as in **(B)** for MDA-MD-231 cells. (Left) Kinetic of extracellular acidification from cells with 5 μM HTH-01-015 (pink curve), 10 μM HTH-01-015 (red curve) or vehicle (blue curve). (Right) Glycolytic parameters evaluation from the experiments on the *left*. Graph shows cells with 5 μM HTH-01-015 (pink bar), 10 μM HTH-01-015 (red bar) or vehicle (blue bar). All values were normalized to the corresponding protein concentration. **(A–C)** ECAR average ± SD from three independent experiments, *p < 0.05. **(D)** ATP levels from MCF-7 cells treated 4 h with 5 μM HTH-01-015 (pink bar), 10 μM HTH-01-015 (red bar) or vehicle (blue bar). Also, all groups were incubated with 1 ug/ml oligomycin A. Results were expressed as a percentage relative to the control group. The results are representative of two independent experiments (*n* = 3). Each bar represents the mean ± S.D, *p < 0.05.

Alterations in mitochondrial metabolism are usually accompanied by glycolysis regulation, allowing energy balance (27). To test this, we measured ECAR, which reflects the rate of lactic acid production by glycolysis. We found that the cytosolic NUAK1 did not significantly affect the glycolytic rate in MCF-7 cells (Figure 5A). However, NUAK1 inhibition significantly decreased their glycolytic capacity (Figure 5B). Although there was a small reproducible effect on the glycolytic rate, it was not significant. To confirm the cytosolic NUAK1 involvement in the glycolytic capacity, we evaluated it in MDA-MB-231 cells. We also found that NUAK1 inhibition significantly decreases glycolytic capacity (Figure 5C), suggesting that the cytosolic NUAK1 maintains these metabolic capacities. Since NUAK1 inhibition affected both mitochondrial and glycolytic capacity, we evaluated whether the ATP level remains balance in NUAK1-inhibited cells. We found that NUAK1 inhibition did not affect ATP in MCF-7 cells under normal conditions, where neither mitochondrial nor glycolytic functions were challenged (Figure 5D). To redirect cellular metabolism to glycolysis, we inhibited the mitochondria with oligomycin. Figure 5D shows that under this condition, HTH-01-015 significantly decreased cellular ATP, without affecting cell viability (data are not shown). Thus, associated with the decrease of glycolytic capacity, NUAK1 inhibition decreases cell energy in a condition of metabolic redirection from OXPHOS to glycolysis. Thereby, our findings suggest that cytosolic NUAK1 keeps ATP balance by maintaining the glycolytic capacity.

### Nuclear NUAK1 Plays a Role in the Glycolytic Switch

**Figure 6:**
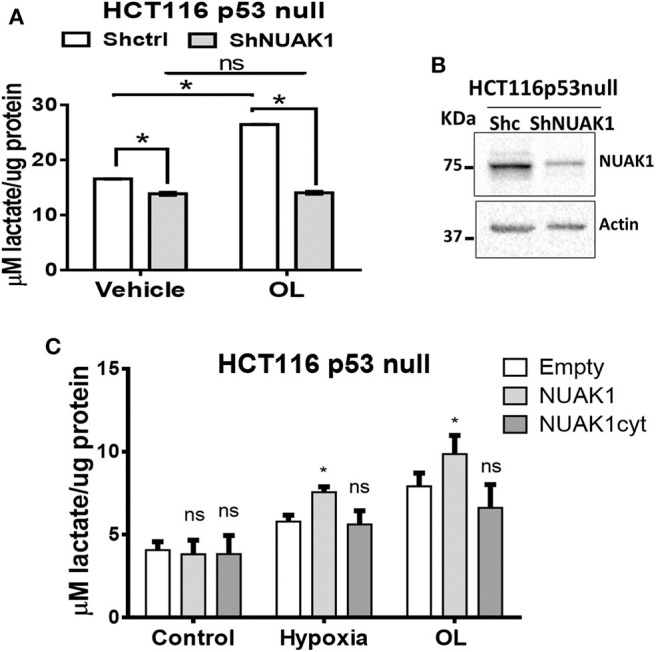
Nuclear NUAK1 allows glycolysis switch in HCT116 p53 null cancer cells. **(A)** Lactate production evaluation in HCT116p53null cells transfected with shRNA NUAK1 (gray bar) and shRNA scramble as control (white bar). Extracellular lactate was evaluated under normal and 1 ug/ml oligomycin treatment conditions. The results were normalized to the corresponding protein concentration and each bar represents the mean ± S.D (*n* = 3), *p < 0.05. **(B)** Western blot showing the NUAK1 silencing efficiency. Actin was used as the loading control. **(C)** Lactate production evaluation in HCT116p53null cells expressing FLAG-NUAK1 WT (gray bar) or FLAG-NUAK1cyt mutant (dark gray bar). Empty vector was used as the control group (white bar). Extracellular lactate was evaluated under normal condition, after 24 h of hypoxia or 24 h of 1 ug/ml oligomycin treatment. The results were normalized to the corresponding protein concentration and each bar represents the mean ± S.D (*n* = 3), *p < 0.05.

Because of the low nuclear expression of NUAK1 in MCF7 cells, we could not discard that this nuclear NUAK1 is responsible for the small reproducible but not significant effect on the glycolytic rate in these cells (see Figure 5B). To evaluate it, we analyzed cells with high nuclear NUAK1 expression. Between the HeLa and the HCT116 p53-null cells, we choose the HCT116 p53-null cells because endogenous NUAK1 is only detected in the nucleus (19). In addition, we used the HCT116 p53-null cell model because NUAK1's role in cell survival has been related to the regulation of the p53 transcription factor (28), which is known to affect glycolysis (29). According to a p53-independent effect, shRNA-mediated knock-down of NUAK1 inhibited HCT116 p53-null cell survival under serum deprivation (data not shown). To directly evaluate an effect on glycolysis, we analyzed NUAK1-dependent lactate production. We found that NUAK1 knock-down (Figure 6A) decreased lactate production in HCT116 p53-null cells under basal conditions (Figure 6B) and blocked their metabolic switch to glycolysis under mitochondrial inhibition (Figure 6B). These results suggested that cells required nuclear NUAK1 for lactate production and glycolytic switch. Accordingly, wild type NUAK1, but not the cytosolic NUAK1, significantly increased lactate production under condition of mitochondrial inhibition by hypoxia or the oligomycin inhibitor in HCT116 p53-null cells (Figure 6C). Thus, nuclear NUAK1 increases glycolysis and is essential for the success of the glycolytic switch.

## Discussion

Our studies indicate that NUAK1 plays a role in the maintenance of glycolytic and respiratory capacities of cancer cells, suggesting that it affects the metabolic state and adaptation of tumors during cancer progression. Also, they suggest that the metabolic outcome depends on the NUAK1 subcellular distribution.

Constant ATP supply is essential for almost all cellular processes, including biomolecules synthesis, cytoskeleton remodeling or signaling phosphorylation (30). In this work, we found that the cytosolic NUAK1 upregulates mitochondrial ATP production, likely by inducing ATP synthase activity. Complete glucose oxidation coupled to TCA cycle and oxidative phosphorylation defines cancer cells susceptibility to apoptosis (31). Accordingly, NUAK1 promoted cell survival and inhibited apoptosis (14, 15); therefore, NUAK1's role in complete glucose oxidation by increased mitochondrial activity could also contribute to tumor viability. However, NUAK1 could also promote cancer cell survival under glucose deprivation (15). Other pathways than glucose oxidation could generate mitochondrial ATP, such as lactate metabolism, glutaminolysis, or fatty acid oxidation (30). Thus, NUAK1 may exert a more integrative regulation for the use of available substrates.

Our data also showed that cytosolic NUAK1 maintains and increases maximal mitochondrial respiration, suggesting that it increases the working capacity of the respiratory chain. Levels of expression of the respiratory complexes are usually associated with the working capacity of the respiratory chain. Because we could not detect any NUAK1-dependent increase in the respiratory complexes nor mitochondrial volumen, NUAK1's effect may be due to increased substrate availability. Nevertheless, we could not discard that NUAK1-dependent phosphorylation of respiratory complexes is responsible for an increase in the respiratory chain activity. Many kinases localize in the mitochondria and affect the mitochondrial function (32). By bioinformatics analysis, NUAK1 does not contain a typical mitochondrial localization signal. However, as reported for other kinases, NUAK1 may form part of a protein complex for translocation into the mitochondrial matrix space (32). This possibility deserves future studies.

Oxidative cells have high anabolic metabolism due to high protein and nucleotide biosynthesis, maintaining high mitochondrial biomass and activity (33). We were unable to find NUAK1 overexpression-induced changes in the mitochondrial volume; however, we cannot exclude that sustained NUAK1 overexpression, common in many cancers, could affect it. Studies have shown that within the heterogeneous cell population of a tumor, oxidative intratumoral cells are the most proliferative, invasive and resistant to chemotherapy and radiotherapy (34, 35). Thus, NUAK1-dependent metabolic effects may explain the aggressiveness of cancers associated with abnormal NUAK1 expression.

Cell energy remains balanced after mitochondrial inhibition due to the increase in glycolysis (27). When the mitochondria activity was pharmacologically inhibited, NUAK1-inhibited cells were unable to maintain ATP levels, indicating that NUAK1 maintains glycolytic ATP levels. However, we cannot discard some contribution from the glutaminolysis pathway. Because glutamine was present in all our experimental conditions, it is possible that this substrate is used as an alternative energy source, compensating for defects in OXPHOS through mitochondrial substrate-level phosphorylation (mSLP) (36–38).

Previous research showed that NUAK1 suppresses glucose uptake by negatively regulating insulin signaling and glycogen storage in the normal oxidative muscle (39). On the contrary, our data propose that the cytosolic NUAK1 maintains glycolytic capacity and the glycolysis-associated cell energy in the abnormal genetic and metabolic context of cancer. Glycolytic capacity may reflect increased activity of enzymes and more efficient expression of alternative isozymes, allowing cells to confront harsh conditions, such as hypoxia (27, 40). The four key points that raise the glycolysis rate are glucose import, hexokinase, phosphofructokinase, and lactate export (40). Several reports describe an increase in the expression of glycolytic enzymes in cancers. In particular, at least one isozyme catalyzing each of the four key points is elevated in human tumors (40). Our studies suggested that nuclear NUAK1 is necessary for the cellular glycolytic switch and the increase of extracellular lactate in a p53-null context. It was recently demonstrated that nuclear NUAK1 promotes spliceosome activity and regulates RNA synthesis (20). Thus, nuclear NUAK1 may transcriptionally affect the expression of enzymes controlling key points of glycolysis. Whether the effect of nuclear NUAK1 changes when p53 is present remains undefined.

It was shown that NUAK1 downregulation dramatically declines HEPG2 cells' tolerance to glucose starvation-induced hypoxia (41). Because metabolic changes in cancer cells are balanced between glycolysis and oxidative metabolism (27), the study indicated that NUAK1 keeps cells metabolically prepared to face microenvironmental energetic adversities. Accordingly, our studies suggest that NUAK1 promotes and maintains both the glycolytic and the oxidative phenotypes. Cytosolic NUAK1 affected both, the maximum rate of glycolysis and mitochondrial respiration. The maximum rate referred to the “metabolic capacity” of cells to respond to an acute increase in energy demand (27).

We found that NUAK1 downregulation disrupts mitochondrial morphology. The ring-shaped mitochondria structures induced by NUAK1 inhibition are consistent with those known as “donut” shaped. Donut morphology appears after inhibition of respiratory chain function and under chemical uncoupling (42, 43) and involves the increase of mitochondrial calcium capture and mitochondrial ROS (mtROS) (43) and have pathophysiological significance (44). NUAK1 has been proposed as a key facilitator of the adaptive antioxidant response in colon cancer, playing a protective role against high oxidative stress (26). We have previously reported that oxidative stress retains NUAK1 in the cytosol (19). Although additional studies are needed, the increase of oxidative stress under NUAK1 inhibition may be responsible for the donut-shaped mitochondria.

Liu et al. (16) showed that NUAK1 expression was essential for the development of oncogenic MYC processes, such as maintaining ATP levels, glucose metabolism, TCA cycle, and oxidative phosphorylation. Some of our findings could be due to an effect of NUAK1 downstream of an oncogenic MYC context; however, addressing this possibility requires a detailed molecular study. Still, because NUAK1 protected cells from oncogenic MYC-induced metabolic stress and energy collapse, NUAK1 is also likely downstream of other oncogenes-induced metabolic stress, the hallmark of any cancer.

In summary, our findings show an association between metabolic NUAK1 functions and its subcellular distribution. We associated nuclear NUAK1 with the promotion of glycolysis. NUAK1 has been described as a predominantly nuclear protein in some cancer cells, where it promotes spliceosome activity and regulates RNA synthesis (20). Thus, glycolysis alterations could be an outcome of those NUAK1 nuclear functions. On the other hand, we associated the cytosolic NUAK1 with the maintenance of cellular ATP levels, suggesting that it increases ATP mitochondrial production under normal conditions. However, it can still maintain ATP from glycolysis source under mitochondrial dysfunction, without discarding some potential contribution of mSLP. NUAK1 showed different cell distribution in cancer samples, where cytosolic NUAK1 seems to be relevant in late-stages of cancer (6, 8, 10). Thereby, NUAK1 cell location could be relevant for metabolic adaptation along with tumor progression. Therefore, screening NUAK1 cell distribution in cancer tissues could help elucidate the metabolic state of tumors. Further studies could shed light on the molecular mechanisms associated with the identified metabolic NUAK1 functions and their implications on cancer cell metabolic adaptation during tumor progression.

## Data Availability Statement

The raw data supporting the conclusions of this article will be made available by the authors, without undue reservation.

## Author Contributions

EE, AE, and AC contributed to conception and design of the study. EE contributed to acquisition and analysis of the most data of this work, performed the statistical analysis, and wrote the first draft of the manuscript. MM took part of the microscopy images acquisition, seahorse assays, and performed mitochondria morphology analysis. AE programed and monitored seahorse and microscopy experiments. AC and RP wrote the final draft of the manuscript. AE, AC, and RP are the principal investigators of the FONDECYT grants that funded this work. All authors contributed to manuscript revision, read and approved the submitted version.

## Conflict of Interest

The authors declare that the research was conducted in the absence of any commercial or financial relationships that could be construed as a potential conflict of interest.
